# Recent Progress in Ophthalmology

**Published:** 1895-06

**Authors:** Frank C. Todd

**Affiliations:** Fort Worth, Texas


### Department of Ophthalmology.

### Recent Progress in Ophthalmology.

BY FRANK C. TODD, M. D., FORT WORTH, TEXAS.

OCULAR GYMNASTICS.—Hobby (*Amer. Jour. of Ophthal.*, June, 1894), advises the practice of muscular exercise in the treatment of strabismus and heteropheria. He produces successive contractions of the weak muscle, for a time just short of fatigue; at first from thirty to one hundred contractions, increasing the number as the patient becomes able to stand more work. He also exercises in a like manner the ciliary muscle, by requiring the patient to look at a distant small object and then at a small near object, securing accurate accommodation each time before changing.

Co-ordination exercise is practice by having the patient look at a distant object and then at a near one, using the same care to secure perfect accommodation, and repeating the changes as often as accurate accommodation will permit. Co-ordination should be practiced at different distances and angles, and the patient should wear a full correction of refractive errors while undergoing the exercise.

---

RETINOSCOPY.—Randall, Wurdemann and Jackson (*Oph Record* IV, 6), read papers before the A. M. A., in San Francisco, on the subject. All agreed that skiascopy was a very valuable test in the determination of errors of refraction, and that no refraction case should be prescribed for until this test had been made. Dr. Randall works at a distance of three or more meters from his patient with an undilated pupil. He thus does not have to make any theoretical allowance for distance, as he does not examine the extra visual zone where most invariably the refraction is different from that of the visual zone. Dr. Jackson who makes his examination at a distance of less than a meter with a dilated pupil, excludes in his examination shadows not appearing in the visual zone, and likes the method better as he can better discriminate the small variations from the normal in small areas of the pupil. These gentlemen all recommended the application of every method at hand in determining errors of refraction in all cases, advising oculists to beware of the inaccurate results obtained by the use of the test lenses alone.

---

ASTIGMIA, NOT ASTIGMATISM.—Martin (*Ann. D'Occel.*, CXIII, 3), gives his reasons why *astigmia* should be substituted for *astigmatism*. Rev. Dr. Whewel who invented this latter word, meant to describe a condition in which rays of light emanating from a luminous point penetrate an eye whose cornea or lens has a greater refraction in one direction than in the perpendicular direction, in which case they do not form an image of a point on the retina, but figures in the form of lines, ovals, or rounded spots.

Whewel, in choosing the substantive to enter into the composition of the word which he wished to form, committed an error: —— ——* does not mean a point in the sense in question, but signifies puncture. Stigmata originates from this word and is commonly used in speaking of the wounds made in the hands and feet of Christ by the nails which held him to the cross. To designate a luminous point, one should have recourse to ———* —, which means the mathematical point. This word requires the termination *ia*, which is found in such words as aphonia.

Dr. Martin mentions that he consulted eminent Greek scholars before recommending this word and received their approval. Among these were M. Ovré, professor of the Bordeaux Faculty of Letters.

---

TUBERCULOSIS INFECTION OF THE EYE.—Bach (*Archives of Ophthalmology* XXIV. 1), describes the manner in which tuberculosis shows itself in the eye, and he lays stress on the following points: 1. Tuberculosis of the eye is by no means a rare affection. 2. All points of the eye may be attacked. 3. It plays a particularly important role in the diseases of the uveal tract. 4. The eye disease may be the only and earliest manifestation of the tuberculosis infection.

---

TUBERCLE OF THE IRIS.—Sandford (*Ophthal. Review*, May, 1894), reports three cases. The first two examples of iritic tubercular deposits occurring at the same time with incipient tuberculosis of the lungs. The other case was one of primary iritic tuberculosis, the suspensory ligament, cilliary region and apparently the lens itself being infiltrated. The case occurred in a child five years old having a history of tuberculosis. No other organ suffered from tuberculosis. The anterior chamber was half filled with the tuberculosis. The eye was enucleated, and eight years afterwards the child was still living.

---

CONSIDERATIONS ON THE CORTICAL VISUAL CENTER.—Vialet (*Rec. d' Ophthal.*, June, 1894), reports cases which seem to confirm the theory that a circumscribed lesion of the internal surface of the occipital lobe may cause persistent hemianopsia, and to justify the localization of the visual center of perception in the three convolutions of this internal surface. The cortical zone in which the visual fibers terminate is not circumscribed as hitherto

---

*The Doctor used the Greek words, and as we have no Greek characters in the office, the words are necessarily omitted.

believed by Henschew, who locates it exclusively in the calcarine fissure.

---

CHANGES IN THE CILIARY BODY FOLLOWING PUNCTURE OF THE ANTERIOR CHAMBER. A STUDY OF THE INTERCHANGE OF FLUIDS IN THE EYE, AND THE FORMATION OF FIBRINE IN THE AQUEOUS HUMOR. Greef. (*Archiv. Oph.*, XXIV, 1).—When the anterior chamber of an animal is punctured, the aqueous humor which escapes is clear, and does not coagulate. Microscopic examination reveals no fibrine. Chemical examination shows no albumen. A second puncture causes a clear aqueous humor to escape, which coagulates immediately, and is found to be rich in albumen and fibrine. We conclude that there are brought about abnormal conditions which make it possible for substances to pass from the lymph into the aqueous humor that in the normal secretions of aqueous humor are held back.

The aqueous humor was evacuated from rabbits' eyes, which were *enucleated* at various intervals thereafter and examined microscipically. Immediately after the puncture, vesicles appear in the ciliary processes, at first small and flat elevations of the epithelium without coagulated contents, and without displacement of the epithelium. These vesicles grow large, and burst, evacuating into the post chamber. Recovery takes place by a healing of the epithelium, and after six days no trace of vesicles remain.

The albuminous substances, which are normally retained, pass out of the serum and form fibrine as soon as the epithelium is elevated.

---

SEPTIC IRIDOCHOROIDITIS CONSECUTIVE TO A UTERINE HEMORRHAGE.—Valude (*Ann. D'Occel.*, CXII, No. 3) reports a typical purulent case coming on the second day after the commencement of an uterine hemorrhage. There were no signs of infection present, the discharge was odorless, and there was no pain.

The cornea and conjunctiva were intact, and there had been no traumatism to the eye. Valude gives his theory as to the cause of the ocular inflammation.

The infection of the deep membranes of the eye is clearly connected, by relation of cause and effect, to the metrorrhœa. The uterus mucous membrane, lacerated by numerous vascular ruptures, served as an entrance of an infectious germ, which proceeded to develop in the ocular media, the uterus, however, remaining free from infection.

TREATMENT OF TRACHOMA.—Scott (*Ophthalmic Record*, IV, 8) read a paper on this subject before the British Medical Association, giving his experience in the treatment of twenty-six cases with chloride of mercury, and the same number with the cyanide of mercury. He found that they were equally effective as to the length of time necessary for a cure, but he favored the cyanide as more agreeable to the patient. He used the cyanide in a four per cent. solution, once a day, applying it to the everted lids, and allowed the patient to use, three times a day, one-fourth per cent solution, dropping it into the conjunctival sac.

He did not favor surgical treatment, nor the use of silver nitrate.

In the discussion, Dr. Knapp said that there were two forms of trachoma. In one form, the trachoma bodies are numerous, and some of them large, while inflammation is slight. These cases should be treated by expression by means of the roller forceps. The inflammatory form, by silver nitrate, copper sulphate, or bichloride.

---

TETANUS FOLLOWING A PENETRATING WOUND OF THE EYE.—Fromaget and Cabannes (*Annales D'Oculistique*, CXII, 1) report one of these rare cases. The patient received a penetrating wound of the eye from the spark from a blacksmith's anvil, causing the eyeball to be emptied of its contents. Four days after the injury, the eye commenced to suppurate, and in four days after suppuration had begun, symptoms of tetanus manifested themselves, causing death in a few days, despite treatment. A peculiar incident in the course of the disease was the absolute fixation of the healthy eye, suggesting ophthalmoplegia.

---

FORMOL AS A HARDENING AGENT.—Marshall (*Ann. D'Oc.*, CXII, 1) has used formol in ten per cent. solutions for hardening the eyeball; after twenty-four hours, it is possible to make sections. The eyeball seems in a fresh condition, the cornea and lens are transparent, and the iris normal. Blood or pus is not changed. The vitreous is unchanged, and both retina and choroid remain *in situ*.

---

THE SIGNIFICANCE OF EYE SYMPTOMS IN KIDNEY DISEASE, *With Some Remarks on the Necessity of a More Rigid Enforcement of the Law Regulating Medical Practice.*—Van Fleet (*N. Y. Med. Journal*, May 11, 1895), points out the advisability of ophthalmoscopic examinations in certain constitutional diseases, and cites several cases which have occurred in his practice where a diagnosis of Bright's disease had been made by the discovery of retinal trouble, before any other symptoms severe enough for the patient to consult a physician had manifested themselves.

He also advised the repeated examination of the urine, in doubtful cases of optic neuritis. Patients not infrequently consult the oculist for relief of headaches, dizziness, etc., when a careful examination fails to reveal any refractive trouble, but the ophthalmoscope, showing optic neuritis, discloses for the first time the cause of the symptoms to be uræmic poisoning.

In addition to inflammatory conditions affecting the back of the eye, as the result of kidney disease, we have also hemorrhages, which are not apparently due to either neuritis or retinitis. This is a condition analagous to apoplexy, and due to a weakened or diseased condition of the blood vessels, occasioned by the deficient elimination of deleterious material from the blood. The same thing can occur as a result of rheumatism, or gout. Hemorrhage into the retina, as a result of atheroma of the vessels in old age, is a grave condition, inasmuch as it often precedes cerebral hemorrhage from the same cause.

If we wish to prolong life in Bright's disease, and prevent blindness, we must begin early. Spectacles will not help the disease, though patients are often fitted by opticians, who prescribe glasses for dimness of vision, whatever the cause, making no ophthalmoscopic examination, and the patient goes on from bad to worse in utter ignorance of his danger.

It should be, he thinks, a criminal offense for any one to prescribe a medicine, a mechanical appliance, or any substance directed to the cure or relief of disease or deformity, unless he shall first comply with the laws governing medical practice, and be thoroughly qualified.

In conclusion, he bespeaks the co-operation of physicians generally, against the "refracting optician," for the benefit of the confiding public, which delights in being humbugged.

